# Immune checkpoint inhibitors combined with tyrosine kinase inhibitors or immunotherapy for treatment-naïve metastatic clear-cell renal cell carcinoma—A network meta-analysis. Focus on cabozantinib combined with nivolumab

**DOI:** 10.3389/fphar.2022.1063178

**Published:** 2023-03-03

**Authors:** Maciej Niewada, Tomasz Macioch, Magdalena Konarska, Aneta Mela, Adam Goszczyński, Bogusława Przekopińska, Karolina Rajkiewicz, Piotr Wysocki, Maciej Krzakowski

**Affiliations:** ^1^ Department of Experimental and Clinical Pharmacology, Centre for Preclinical Research and Technology (CePT), Medical University of Warsaw, Warsaw, Poland; ^2^ HealthQuest Sp. z.o.o, Warsaw, Poland; ^3^ Ipsen Polan Sp. z.o.o, Warsaw, Poland; ^4^ Department of Oncology, Jagiellonian University-Collegium Medicum Hospital, Cracow, Poland; ^5^ Department of Lung Cancer and Chest Tumors, the Maria Sklodowska-Curie National Research Institute of Oncology, Warsaw, Poland

**Keywords:** renal cancer, immunotherapy, tyrosine kinase inhibitors, indirect comparison, network meta-analysis

## Abstract

**Introduction:** The combination of immunotherapy and targeted therapy is currently marking a new era in the treatment of renal cancer. The latest clinical guidelines recommend the use of drug combinations for the first-line treatment of advanced renal cancer. The aim of this review is to compare the efficacy of combined cabozantinib + nivolumab therapy with other immune checkpoint inhibitors combined with tyrosine kinase inhibitors or monoclonal antibodies blocking the CTLA-4 (cytotoxic T cell antigen 4) in the first-line treatment of metastatic clear-cell renal cell carcinoma (RCC).

**Methodology:** A systematic literature search was carried out in the PubMed and EMBASE databases. Randomized controlled trials (RCTs) on therapies recommended by the latest EAU and ESMO guidelines for treatment-naïve metastatic RCC (i.e., lenvatinib + pembrolizumab, axitinib + pembrolizumab and nivolumab + ipilimumab) were searched. A network meta-analysis (NMA) was performed for data synthesis. The methodology of included RCTs was assessed using the Cochrane RoB two tool. The data were analyzed in the overall population as well as in risk subgroups defined according to the International Metastatic Database Consortium (IMDC) i.e., patients with a favorable and intermediate or poor prognoses. The most recent cut-off dates from included studies were analyzed.

**Results:** Four RCTs (CheckMate 9 ER, KEYNOTE-426, CLEAR and CheckMate 214) were included in the review. No studies directly comparing cabozantinib + nivolumab with any of the drug combinations included in this review were available. NMA showed that cabozantinib + nivolumab was superior compared to axitinib + pembrolizumab and nivolumab + ipilimumab in all analyzed comparisons (overall population and IMDC risk subgroups), both in terms of overall survival and progression-free survival (PFS). The advantage of cabozantinib + nivolumab was statistically significant only for PFS when compared to nivolumab + ipilimumab in the overall population. The results for the comparison of cabozantinib + nivolumab with lenvatinib + pembrolizumab showed numerical superiority of lenvatinib + pembrolizumab combination in terms of overall survival, but none of the results were statistically significant. The advantage of lenvatinib + pembrolizumab over cabozantinib + nivolumab in terms of PFS was statistically significant in the overall and favorable prognosis population.

**Conclusion:** Inclusion of the most recent cut-off data from CheckMate 9 ER did not affect the role of the cabozantinib + nivolumab combination for treatment-naïve metastatic RCC. Cabozantinib + nivolumab is an effective therapeutic option for the first-line treatment of advanced renal cancer that is recommended both in the latest European and American guidelines for all IMDC risk groups.

## Introduction

Renal cell carcinoma (RCC) is the third most common malignant neoplasm of the genitourinary system. This cancer accounts for 5% of all malignant neoplasms in men and 3% in women (Bray et al., 2018). Clear-cell RCC originates in the cortex of the kidney and accounts for 80% of all cases of renal cancer. The highest incidences of RCC are recorded in Western Europe and the United States. Overall, there has been a significant increase in the incidence of RCC annually in the last decades both globally and in Europe (Padala et al., 2020). In most cases, kidney cancer is asymptomatic and the patient has no prodromal symptoms. Currently, most of RCC cases are diagnosed based on the imaging performed for other reasons (Padala et al., 2020).

The combination of immunotherapy and targeted therapy now marks a new era in the treatment of renal cancer. In the latest European and American guidelines, the recommended first-line treatment for advanced renal cancer is a combination of drugs, including combinations of tyrosine kinase inhibitors (TKI) or monoclonal antibodies blocking the CTLA-4 (cytotoxic T cell antigen 4) with anti-PD1 or PD-L1 monoclonal antibodies (Bedke et al., 2021; Powles et al., 2021; Motzer et al., 2022a). Numerous clinical trials have demonstrated the superiority of combination therapy in terms of increasing overall survival (OS) and progression-free survival (PFS) compared to sunitinib monotherapy (Motzer et al., 2018; Rini et al., 2019; Choueiri et al., 2021; Motzer et al., 2021). The available combinations may have different safety and efficacy profiles; however, no studies have directly compared the various combination therapies.

To date, several systematic reviews have summarized the data reported in clinical trials evaluating first-line combination therapies for metastatic RCC (Cattrini et al., 2021; Ciccarese et al., 2021; Mori et al., 2021; Quhal et al., 2021; Riaz et al., 2021; Bosma et al., 2022; Nocera et al., 2022). However, in most of the published reviews, the authors presented the results only in comparison with sunitinib monotherapy and not including comparisons between the included combination therapies. The exceptions are three systematic reviews (Riaz et al., 2021; Nocera et al., 2022 and Bosma et al., 2022) in which the authors performed a network meta-analysis and presented comparisons for the combination first-line therapies for metastatic RCC.12, 15, 18 However, in all of these reviews, the clinical data for cabozantinib + nivolumab included data from the CheckMate 9 ER study reported for the March 2020 cutoff date (median follow-up of 18.1 months), while more recent data with the June 2021 cutoff (median follow-up of 32.9 months) are already available (Motzer et al., 2022b). Consistently more recent and mature data published for other combinations (i.e., CheckMate 214 - Motzer 2022b) are available (Motzer et al., 2022c). Moreover, the Nocera et al. (2022) review reported only overall population results and did not analyze the results separately for the International Metastatic Database Consortium (IMDC) risk subgroups.15.

The aim of this review is to update on the relative efficacy of combined cabozantinib + nivolumab therapy with other immune checkpoint inhibitors combined with TKIs or monoclonal antibodies blocking the CTLA-4 for the first-line treatment of metastatic clear-cell renal cell carcinoma.

## Methods

### Search strategy and inclusion criteria

The review was performed following Preferred Reporting Items for Systematic Reviews and Meta-Analyses (PRISMA) guidelines (Page et al., 2021). A search for randomized controlled clinical trials (RCTs) in the PubMed and EMBASE databases was performed. The search strategy is presented in Supplementary Material S1. The search process also used references found in primary publications, internet search engines and clinical trial registries. RCTs recruiting patients receiving first-line treatment for advanced renal cancer and evaluating therapies with the following combinations were included: cabozantinib + nivolumab, axitinib + pembrolizumab, lenvatinib + pembrolizumab, and nivolumab + ipilimumab. Article selection was performed independently by two researchers (B.P, K.R.). In the absence of identification of head-to-head comparisons, studies were sought that would enable indirect comparisons to be made. Data on OS and PFS were extracted and analysed. The analysis included both full-text publications and conference abstracts, in which newer data was reported in relation to full-text publications. We excluded any other than RCT studies (i.e., retrospective, non-controlled, non-randomized, observational or real-world evidence), as well post-hoc reports or reports on subpopulation other than IMDC risk subgroups (i.e., we excluded results for specific organ metastasis, results in race or region subpopulation *etc.*). We also excluded reports with lack of new data regarding overall survival and progression-free survival as compared to the main publications. No language filter was applied. The last update was carried out on 28/07/2022.

### Data extraction and synthesis

In line with the recommendations presented in the latest European Association of Urology (EAU) and European Society for Medical Oncology (ESMO) guidelines, the following combinations were considered for the subpopulation of patients with a favorable prognosis: cabozantinib + nivolumab, axitinib + pembrolizumab and lenvatinib + pembrolizumab.4,6 In the case of the patients with an intermediate/poor prognosis (as well as in the case of the overall population), the combination of nivolumab + ipilimumab were added to analysis.

RCTs identified in the literature search were assessed using the current Cochrane Risk of Bias tool (RoB, version 2) (Higgins et al., 2021). Data from the identified studies were extracted by one of the authors (B.P.) and entered into a Microsoft Excel spreadsheet, which was then independently checked for correctness by another author (K.R.). The OS and PFS data reported for the latest published cut-off dates for each identified study were extracted. Depending on data availability, data were extracted from both full-text publications and conference abstracts. Data were extracted for both the overall populations included in each study and for the risk subgroups of patients as assessed by IMDC. The results reported for the subgroup of patients with a favorable prognosis and those with an intermediate/poor prognosis were included in this review. In the absence of data for combined population of patients with an intermediate/poor prognosis, a meta-analysis of data for patients in the intermediate and poor risk group was used. Calculations were conducted in Review Manager software (version 5.4). For PFS, the results assessed by the Independent Review Committee (IRC) were taken into account.

Comparisons of the included drug combinations used for the first-line treatment of renal cancer were carried out using a network meta-analysis. For both endpoints (OS and PFS), the results are presented in the form of a HR and a 95% confidence interval (CI). Assessment of the relative effectiveness of the compared drugs was performed using the Bayesian approach (Spiegelhalter et al., 2002). It was assumed that the effect observed in the study (observed HR value) is derived by the probability distribution and that the logHR is given as a normal distribution with a standard deviation consistent with the estimated error in the studies. Due to the small number of studies with similar pairs of compared drugs (all included studies compared with sunitinib monotherapy), a fixed effects model was adopted. A fixed effects model was also adopted because it is in practice (in the absence of loops in the network) equivalent to the Bucher approach (i.e., all connections included in the network connect through one common comparator: sunitinib) (Bucher et al., 1997). In the analysis, non-informative prior distributions were adopted in order to arrive with results based on the observed research results as much as possible. The modeling used the Markov Chain Monte Carlo (MCMC) approach. The code was implemented in JAGS and run from the R program. The results were based on the values ​​of 50,000 iterations (every fifth iteration was taken to reduce the problem with possible correlation of values) with the first 50,000 iterations discarded as “burn-in”. The approach outlined above allows all drug combinations included in the network to be compared. Additionally, for each outcome, we report treatment ranks (probabilities) based on the surface under the cumulative ranking curve (SUCRA) (Salanti et al., 2011). The analysis was performed with the R software (version 4.5.0).

## Results

### Studies included

**FIGURE 1:**
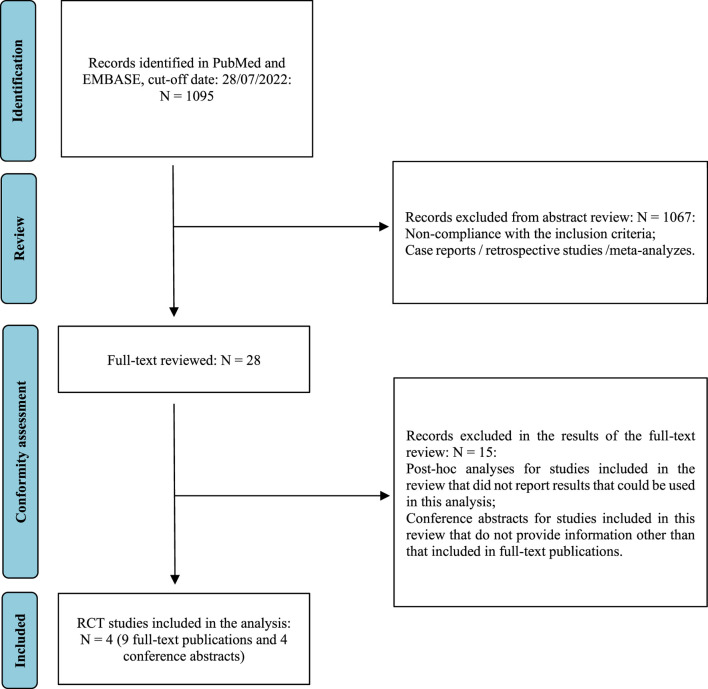
The study selection process (PRISMA) (Moher et al., 2009).

**FIGURE 2:**
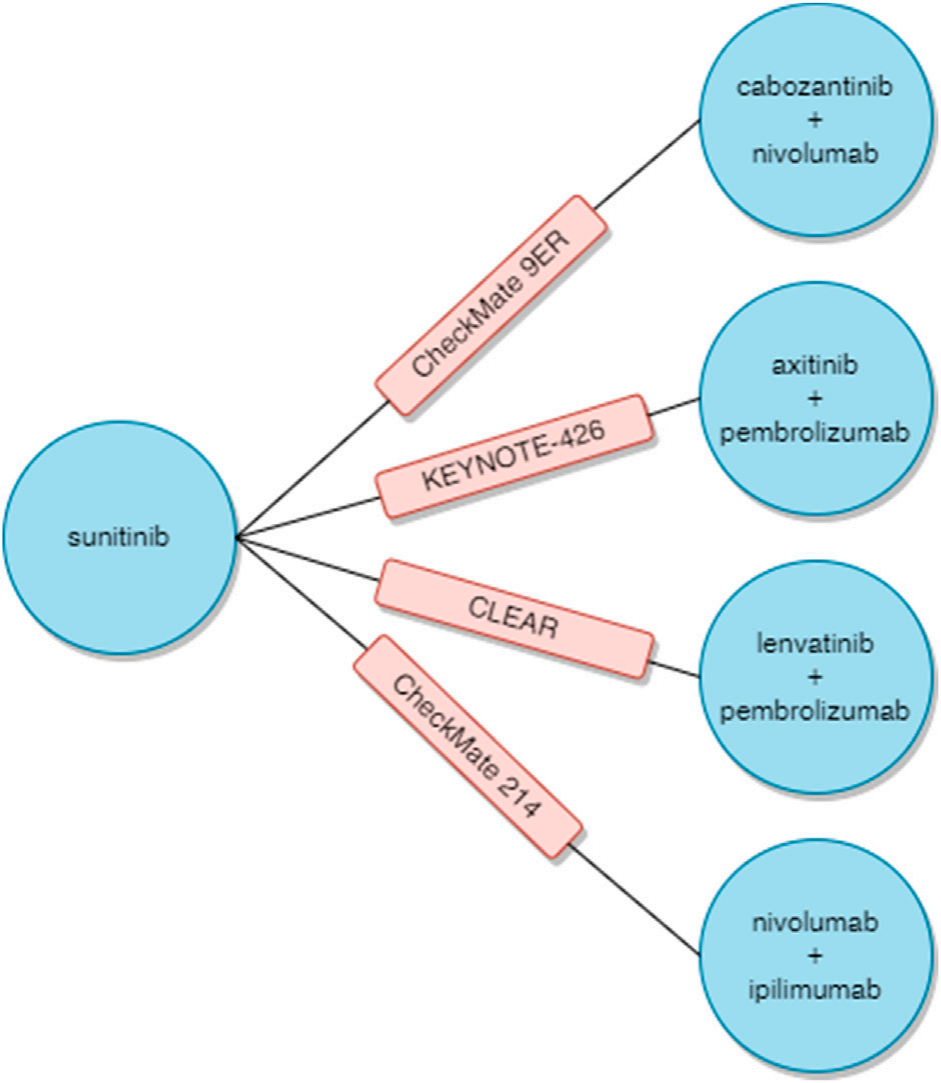
Network analysis.

As a result of the searches in the PubMed and EMBASE databases, 1,095 abstracts were found and were preliminarily assessed. After verifying the relevance of the title and abstract and eliminating replicates, 28 full texts were analyzed in detail to determine if they met the inclusion and exclusion criteria for the study. Finally, the review included four RCTs, described in 13 full-text publications and abstracts: CheckMate 9 ER (cabozantinib + nivolumab vs sunitinib), KEYNOTE-426 (axitinib + pembrolizumab vs sunitinib), CLEAR (lenvatinib + pembrolizumab vs sunitinib) and CheckMate 214 (nivolumab + ipilimumab vs sunitinib). The detailed study selection process is presented in Figure 1. Supplementary Material S2 contains a detailed description of the included studies, while Supplementary Material S3 presents the list of excluded studies and the reasons for exclusion. As no direct comparisons of cabozantinib + nivolumab treatment to other combined therapies were found, indirect comparisons using network analysis were performed. Figure 2 illustrates the network used for comparisons of the OS and PFS results in the overall population. Supplementary Material S4 show the networks used for the subpopulations of patients with a favorable prognosis and those with an intermediate/poor prognosis.

### Risk of bias

**FIGURE 3:**
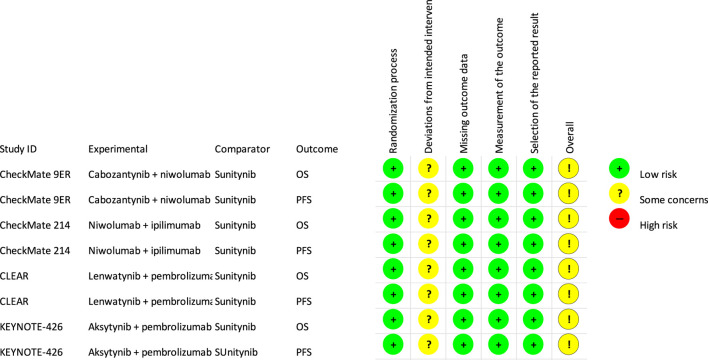
Risk of bias (RoB, version 2) (Higgins et al., 2019).

A low risk of bias was obtained for most of the endpoints and for most of the analyzed domains, except for domain of deviations from intended interventions. Thus, we judged to have “some concern” for an overall risk of bias. In detail, in all identified studies, the randomization was carried out correctly with adequate allocation sequence concealment. For both OS and PFS, the results were assessed in the intention to treat (ITT) population and appropriate performance measurement methods were used. All studies had a study protocol with a detailed analysis plan defined in advance. In the domain of deviation from planned interventions, both OS and PFS (in all analyzed studies) were assessed as having some concerns regarding the risk of bias. For all analyzed treatment arms, not all patients randomized to a given group received the study drug, but it should be emphasized that the percentage of patients who did not receive scheduled treatment was negligible, ranging from <1% for the majority of the analyzed treatment arms to 5% for the sunitinib treatment arm in CLEAR study. In addition, all the studies were open and allocation was only partially blinded for both researchers and patients. Nevertheless, it should be noted that, due to the nature of the main assessed endpoint (OS), blinding or its absence is not a concern. Moreover, for PFS in all analyzed studies, the results were assessed by an IRC blinded to allocation to the treatment group. The detailed results for risk of bias are summarized in Figure 3.

### Network analysis results

Supplementary Material S5 summarizes the results extracted from the individual studies included in this review (i.e., the OS and PFS results reported in the identified publications or conference abstracts).

### Overall survival

Both the results estimated for the overall population and the IMDC risk subpopulations showed no statistically significant differences in HRs for OS between cabozantinib + nivolumab and any of the analyzed drug combinations. Cabozantinib + nivolumab was numerically superior to the included combinations in most of the analyzed comparisons. The exceptions are the results for the comparison of cabozantinib + nivolumab with lenvatinib + pembrolizumab in the case of the overall population, and the subpopulation of patients in the intermediate/poor prognosis group where lenvatinib + pembrolizumab was numerically superior to cabozantinib + nivolumab. Analysis of the subpopulation of patients with a favorable prognosis showed cabozantinib + nivolumab therapy to be the best available treatment (see Table 4). In the overall population and the subpopulation with an intermediate/poor prognosis, analysis showed cabozantinib + nivolumab to be the second best therapy after lenvatinib + pembrolizumab. However, it should be emphasized that the estimated probability value of being the best therapy for cabozantinib + nivolumab was almost equal to that for lenvatinib + pembrolizumab. The detailed results are summarized in Tables 1–4.

**TABLE 1:** Hazard ratios (HR) for overall survival in the overall population.

		Intervention [HR (95% CI)]				
		CAB + NIV	AXY + PEM	LEN + PEM	NIV + IPI	SUN
Comparator	CAB + NIV		1.06 (0.75; 1.40)	0.96 (0.61; 1.34)	1.04 (0.75; 1.35)	1.44 (1.10; 1.81)
	AXY + PEM	0.97 (0.69; 1.29)		0.92 (0.62; 1.26)	0.99 (0.75; 1.24)	1.38 (1.12; 1.65)
	LEN + PEM	1.08 (0.69; 1.5)	1.12 (0.75; 1.53)		1.11 (0.76; 1.49)	1.53 (1.11; 2.00)
	NIV + IPI	0.98 (0.70; 1.27)	1.02 (0.77; 1.28)	0.93 (0.64; 1.25)		1.39 (1.18; 1.61)
	SUN	0.71 (0.54; 0.88)	0.73 (0.59; 0.87)	0.67 (0.48; 0.87)	0.72 (0.61; 0.84)	

The values in each cell represent the relative treatment effect for the intervention on the top when compared to the intervention on the left. Green suggests a relative treatment benefit (light green a nonsignificant benefit, and dark green a significant benefit). Red suggests a relative treatment harm (light red a nonsignificant harm, and dark red a significant harm).AXY, axitinib; CAB, cabozantinib; IPI, ipilimumab; LEN, lenvatinib; PEM, pembrolizumab; NIV, nivolumab; SUN, sunitinib

**TABLE 2:** Hazard ratios (HR) for overall survival in the population with a favorable prognosis.

		Intervention [HR (95% CI)]			
		CAB + NIV	AXY + PEM	LEN + PEM	SUN
Comparator	CAB + NIV		1.23 (0.43; 2.2)	1.26 (0.30; 2.56)	1.02 (0.45; 1.68)
	AXY + PEM	0.95 (0.33; 1.71)		1.08 (0.32; 2.06)	0.88 (0.51; 1.26)
	LEN + PEM	1.01 (0.23; 2.05)	1.12 (0.33; 2.13)		0.93 (0.34; 1.65)
	SUN	1.08 (0.47; 1.80)	1.20 (0.72; 1.74)	1.23 (0.45; 2.18)	

The values in each cell represent the relative treatment effect for the intervention on the top when compared to the intervention on the left. Green suggests a relative treatment benefit (light green a nonsignificant benefit, and dark green a significant benefit). Red suggests a relative treatment harm (light red a nonsignificant harm, and dark red a significant harm).AXY, axitinib; CAB, cabozantinib; IPI, ipilimumab; LEN, lenvatinib; NIV, nivolumab; PEM, pembrolizumab; SUN, sunitinib

**TABLE 3:** Hazard ratios (HR) for overall survival in the population with an intermediate/poor prognosis.

		Intervention [HR (95% CI)]				
		CAB + NIV	AXY + PEM	LEN + PEM	NIV + IPI	SUN
Comparator	CAB + NIV		1.05 (0.61; 1.54)	0.95 (0.51; 1.46)	1.11 (0.66; 1.61)	1.62 (1.01; 2.29)
	AXY + PEM	1.01 (0.59; 1.49)		0.92 (0.58; 1.29)	1.07 (0.78; 1.37)	1.57 (1.23; 1.91)
	LEN + PEM	1.12 (0.60; 1.73)	1.13 (0.71; 1.57)		1.19 (0.79; 1.65)	1.75 (1.21; 2.32)
	NIV + IPI	0.95 (0.56; 1.37)	0.95 (0.70; 1.22)	0.87 (0.57; 1.19)		1.48 (1.24; 1.73)
	SUN	0.64 (0.40; 0.90)	0.64 (0.51; 0.79)	0.59 (0.41; 0.79)	0.68 (0.57; 0.80)	

The values in each cell represent the relative treatment effect for the intervention on the top when compared to the intervention on the left. Green suggests a relative treatment benefit (light green a nonsignificant benefit, and dark green a significant benefit). Red suggests a relative treatment harm (light red a nonsignificant harm, and dark red a significant harm).AXY, axitinib; CAB, cabozantinib; IPI, ipilimumab; LEN, lenvatinib; NIV, nivolumab; PEM, pembrolizumab; SUN, sunitinib

**TABLE 4:** The likelihood of being the preferred treatment option compared to the other treatment strategies probability of being the best treatment (SUCRA) in terms of overall survival.

Intervention	SUCRA (%)
Overall population	
Lenvatinib + pembrolizumab	75.5
Cabozantinib + nivolumab	64.0
Nivolumab + ipilimumab	56.9
Axitinib + pembrolizumab	53.5
Sunitinib	0.1
Subpopulation with a favorable prognosis	
Cabozantinib + nivolumab	56.1
Lenvatinib + pembrolizumab	42.9
Sunitinib	64.8
Axitinib + pembrolizumab	36.2
Subpopulation with an intermediate/poor prognosis	
Lenvatinib + pembrolizumab	77.9
Cabozantinib + nivolumab	63.4
Axitinib + pembrolizumab	61.1
Nivolumab + ipilimumab	47.3
Sunitinib	0.3

### Progression free survival

Both the results estimated for the overall population and the IMDC risk subpopulations showed—in the majority of the analyzed comparisons—superiority in the HR for PFS for cabozantinib + nivolumab compared to the other included combinations (the comparison with nivolumab + ipilimumab in the overall population showed statistically significant differences in favor of cabozantinib + nivolumab). The exceptions are the results for the comparison of cabozantinib + nivolumab with lenvatinib + pembrolizumab. For all analyzed populations, lenvatinib + pembrolizumab was numerically superior compared to cabozantinib + nivolumab and the advantage of lenvatinib + pembrolizumab over cabozantinib + nivolumab was statistically significant in the overall and favorable prognosis population. In all analyzed populations, analysis showed cabozantinib + nivolumab to be the second best therapy after lenvatinib + pembrolizumab. The detailed results are summarized in Tables 5–8.

**TABLE 5:** Hazard ratios (HR) for progression-free survival in the overall population.

		Intervention [HR (95% CI)]				
		CAB + NIV	AXY + PEM	LEN + PEM	NIV + IPI	SUN
Comparator	CAB + NIV		1.23 (0.92; 1.54)	0.70 (0.51; 0.91)	1.55 (1.17; 1.95)	1.80 (1.45; 2.15)
	AXY + PEM	0.83 (0.63; 1.04)		0.58 (0.43; 0.73)	1.27 (1.00; 1.57)	1.47 (1.24; 1.71)
	LEN + PEM	1.45 (1.06; 1.89)	1.76 (1.30; 2.23)		2.23 (1.66; 2.84)	2.58 (2.05; 3.15)
	NIV + IPI	0.66 (0.50; 0.83)	0.80 (0.62; 0.99)	0.46 (0.34; 0.58)		1.17 (0.98; 1.36)
	SUN	0.56 (0.46; 0.67)	0.68 (0.57; 0.79)	0.39 (0.31; 0.48)	0.86 (0.73; 1.00)	

The values in each cell represent the relative treatment effect for the intervention on the top when compared to the intervention on the left. Green suggests a relative treatment benefit (light green a nonsignificant benefit, and dark green a significant benefit). Red suggests a relative treatment harm (light red a nonsignificant harm, and dark red a significant harm).AXY, axitinib; CAB, cabozantinib; IPI, ipilimumab; LEN, lenvatinib; NIV, nivolumab; PEM, pembrolizumab; SUN, sunitinib

**TABLE 6:** Hazard ratios (HR) for progression-free survival in the population with a favorable prognosis.

		Intervention [HR (95% CI)]			
		CAB + NIV	AXY + PEM	LEN + PEM	SUN
Comparator	CAB + NIV		1.08 (0.56; 1.65)	0.59 (0.29; 0.94)	1.40 (0.84; 2.00)
	AXY + PEM	0.99 (0.52; 1.52)		0.56 (0.30; 0.85)	1.33 (0.94; 1.74)
	LEN + PEM	1.85 (0.91; 2.98)	1.91 (1.03; 2.90)		2.49 (1.59; 3.53)
	SUN	0.74 (0.45; 1.07)	0.77 (0.55; 1.01)	0.42 (0.27; 0.59)	

The values in each cell represent the relative treatment effect for the intervention on the top when compared to the intervention on the left. Green suggests a relative treatment benefit (light green a nonsignificant benefit, and dark green a significant benefit). Red suggests a relative treatment harm (light red a nonsignificant harm, and dark red a significant harm).AXY, axitinib; CAB, cabozantinib; IPI, ipilimumab; LEN, lenvatinib; NIV, nivolumab; PEM, pembrolizumab; SUN, sunitinib

**TABLE 7:** Hazard ratios (HR) for progression-free survival in the population with an intermediate/poor prognosis.

		Intervention [HR (95% CI)]				
		CAB + NIV	AXY + PEM	LEN + PEM	NIV + IPI	SUN
Comparator	CAB + NIV		1.48 (0.75; 2.31)	0.80 (0.39; 1.26)	1.61 (0.83; 2.50)	2.19 (1.19; 3.35)
	AXY + PEM	0.73 (0.38; 1.14)		0.54 (0.38; 0.73)	1.10 (0.82; 1.39)	1.50 (1.22; 1.79)
	LEN + PEM	1.37 (0.67; 2.18)	1.89 (1.32; 2.53)		2.06 (1.43; 2.71)	2.80 (2.09; 3.54)
	NIV + IPI	0.67 (0.35; 1.04)	0.93 (0.69; 1.17)	0.50 (0.35; 0.66)		1.38 (1.13; 1.62)
	SUN	0.49 (0.26; 0.74)	0.67 (0.55; 0.81)	0.36 (0.27; 0.46)	0.73 (0.60; 0.86)	

The values in each cell represent the relative treatment effect for the intervention on the top when compared to the intervention on the left. Green suggests a relative treatment benefit (light green a nonsignificant benefit, and dark green a significant benefit). Red suggests a relative treatment harm (light red a nonsignificant harm, and dark red a significant harm).AXY, axitinib; CAB, cabozantinib; IPI, ipilimumab; LEN, lenvatinib; NIV, nivolumab; PEM, pembrolizumab; SUN, sunitinib

**TABLE 8:** The likelihood of being the preferred treatment option compared to the other treatment strategies probability of being the best treatment (SUCRA) in terms of progression-free survival.

Intervention	SUCRA (%)
Overall population	
Lenvatinib + pembrolizumab	99.8
Cabozantinib + nivolumab	73.5
Axitinib + pembrolizumab	51.1
Nivolumab + ipilimumab	24.7
Sunitinib	0.9
Subpopulation with a favorable prognosis	
Lenvatinib + pembrolizumab	98.9
Cabozantinib + nivolumab	50.8
Axitinib + pembrolizumab	46.8
Sunitinib	3.5
Subpopulation with an intermediate/poor prognosis	
Lenvatinib + pembrolizumab	95.6
Cabozantinib + nivolumab	75.5
Axitinib + pembrolizumab	45.9
Nivolumab + ipilimumab	32.9
Sunitinib	0.1

## Discussion

This systematic review updated on relative efficacy of different combination therapies (tyrosine kinase inhibitors or monoclonal antibodies blocking the CTLA-4 combined with immune checkpoint inhibitors) for the first-line treatment of metastatic clear-cell renal cell carcinoma. The combination therapy of TKIs or monoclonal antibodies blocking the CTLA-4 combined with immune checkpoint inhibitors are recommended in the latest European guidelines (EAU and ESMO) for the first-line treatment of metastatic RCC. In the EAU and ESMO guidelines, recommendations regarding the therapies are presented by the prognostic group according to the IMDC scale. In the population of patients with a favorable prognosis, the therapies recommended for use are cabozantinib + nivolumab, axitinib + pembrolizumab, and lenvatinib + pembrolizumab, while in the population of patients with an intermediate/poor prognosis—apart from combination therapies listed for a favorable prognosis—nivolumab + ipilimumab is also recommended. Therefore, in this review, in addition to the results analyzed for the overall population, the results for the sub-populations of patients with favorable and intermediate/poor prognoses are also presented.

The database search identified no studies that directly compare any of the drug combinations included in this review. Therefore, we performed indirect comparisons using a network meta-analysis. All of the studies included in the network were assessed as having some concern regarding the risk of bias. However, it should be emphasized that such an assessment was obtained only in the case of one out of the five domains assessed with the RoB two tool. In the remaining four domains, a low risk of bias was reported for all analyzed studies and all analyzed endpoints. Therefore, the RCT studies included in the network meta-analysis can be considered as high-quality input studies in terms of methodology and presented data.

In this review, the results were analyzed for two endpoints: OS and PFS. OS is a highly significant clinical endpoint—both from the patient’s and the clinician’s perspective—and is a widely accepted measure of benefit that can be easily and accurately assessed. At the same time, it should be emphasized evaluation of survival gain results in larger sample population and longer follow-up to show statistically significant differences between the compared groups. For the CLEAR study, it was noted that there were too few events (deaths) in the subpopulation of patients with a favorable prognosis to carry out a proper analysis. Therefore, the OS results for the subpopulation of patients with a favorable prognosis in the CLEAR study should be interpreted with caution. In oncology studies, in addition to OS (the gold standard), PFS—an endpoint considered as a surrogate—is also very relevant. Currently, PFS is increasingly used as the endpoint of clinical trials for the authorization of new drugs used in advanced malignancies. (EMA/CHMP/27994/2008/Rev.1).

There are several limitations of this analysis. First, it is based on indirect comparisons due to lack of direct head-to-head studies. Indirect comparison may suffer from the biases of observational studies, thus all results should be interpreted with caution (Higgins et al., 2022). Moreover, some of the data used in calculations were derived from abstracts that have not undergone the peer-review process. It should be also noted that although Checkmate 214 trial included patients with favorable, intermediate and poor risk patients, the primary endpoint was reported on intermediate and poor IMDC risk only - subpopulations with relatively better results when compared with sunitinib. This may affect results of analysis in overall population given other included trials covered also favorable risk population. EAU and ESMO guidelines recommend combinations of TKI with anti-PD1 or PD-L1 monoclonal antibodies in the first-line treatment of all mRCC patients, including ones with favorable prognosis. However, specific evidences on the superiority of combined treatment over TKI monotherapy for that subpopulation are less consistent and disputable. Thus, some clinical guidelines (i.e., Polish Society of Clinical Oncology and Polish Urological Association) do not recommend combinations of TKI with anti-PD1 or PD-L1 monoclonal antibodies in favorable prognosis mRCC patients keeping this treatment for patients with intermediate and poor IMDC risk only (Wysocki et al., 2020). Current treatment of mRCC is sequential, thus, combination therapy used early on in first-line therapy of favorable risk patients could affect subsequent management options. Additionally, our findings heavily depend on the validity of the proportional hazards (PH) assumption in the estimation of HR. Preliminary analysis by Marciniak and al. Showed PH assumption to be violated for some comparisons used in this NMA (Marciniak et al., 2022). We decided to present our findings corresponding to previous NMAs based on PH assumption. However, they should be interpreted cautiously; we believe a more robust NMA based on the full Kaplan–Meier curves and methods to explore and if needed adjust for non-proportionality of hazard should further verify and enrich the comparative effectiveness findings.

In the overall population, treatment with cabozantinib + nivolumab was ranked second in terms of probability of being the best treatment (SUCRA) as measured by PFS or OS. Similar conclusions have also been presented for PFS in the previously published systematic reviews that were based on earlier cut-off data—see Table 9. However some previously published systematic reviews ranked treatment with cabozantinib + nivolumab the best treatment as measured by OS (Quhal et al., 2021; Bosma et al., 2022; Nocera et al., 2022). There are several issues that may cause some differences in rankings. First of all, attention should be paid to up-to-date data used in those previously published systematic reviews. All of them used interim data from the CheckMate 9 ER study reported for the March 2020 cutoff date (median follow-up of 18.1 months), while in our review recent data with the June 2021 cutoff (median follow-up of 32.9 months) were used. We used more mature and latest available data for all RCTs to those used in previously published systematic reviews (i.e., median follow-up of 67.7 months data from CheckMate 214, while most recently published review by Bosma et al. used median follow-up of 55 months) (Bosma et al., 2022). We believe that also differences in scope of the reviews i.e., number of interventions included, may cause some differences in NMA results. Following recommendations presented in the latest European Association of Urology (EAU) and European Society for Medical Oncology (ESMO) guidelines, we considered for the subpopulation of patients with a favorable prognosis: cabozantinib + nivolumab, axitinib + pembrolizumab and lenvatinib + pembrolizumab. For intermediate/poor prognosis patients (as well as in the case of the overall population), the combination of nivolumab + ipilimumab were added to analysis. Apart of this combination Bosma et al. considered also avelumab + axitinib and atezolizumab + bevacizumab (Bosma et al., 2022). The living systematic review performed by Riaz et al. includes also monotherapies with sorafenib, cabozantinib, tivozanib and pazopanib (Riaz et al., 2021). It should be mentioned that only Bosma et al. and Riaz et al. used SUCRA for ranking probability of being the best treatment. Nethertheless, our results are similar to previously published reviews both in the overall population and in subpopulations of patients with a favorable or intermediate/poor prognosis—see Table 9.

**TABLE 9:** Rank order (SUCRA) of 1L aRCC combination therapies for OS and PFS across eligible NMAs.

Endpoint	NMA	Ranking [ranking probability (%)]			
		CAB + NIV	AXY + PEM	LEN + PEM	NIV + IPI
OS overall population	current study	2 (64.0%)	4 (53.5%)	1 (75.5%)	3 (56.9%)
	Riaz et al. (2021)	2 (82.35%)	3 (80.22%)	1 (83.0%)	4 (79.35%)
	Quhal^11^ ^a^	1 (75.73%)	3 (64.96%)	2 (67.46%)	4 (63.58%)
	Nocera^15^ ^a^	1 (77%)	3 (57%)	2 (63%)	4 (53%)
	Bosma et al. (2022)	1 (82%)	3 (68%)	2 (72%)	4 (66%)
OS IMDC favorable risk population	current study	1 (56.1%)	4 (36.2%)	2 (42.9%)	n.e
	Riaz et al. (2021)	3 (60.24%)	6 (37.34%)	7 (31.49%)	4 (53.54%)
	Quhal^11^ ^a^	2 (55,27%)	5 (45.39%)	6 (43.43%)	3 (52.09%)
	Bosma et al., 2022)	2 (62%)	5 (40%)	6 (33%)	3 (56%)
OS IMDC intermediate or poor risk population	current study	2 (63.4%)	3 (61.1%)	1 (77.9%)	4 (47.3%)
	Riaz et al. (2021)	1 (82.03%)	3 (69.56%)	2 (73.14%)	4 (65.1%)
	Quhal^11^ ^a^	2 (83.52%)	5 (31.13%)	1 (86.53%)	3 (49.24%)
	Bosma - intermediate Bosma et al. (2022)	3 (60%)	1 (78%)	4 (56%)	2 (71%)
	Bosma - poor Bosma et al. (2022)	2 (80%)	5 (42%)	1 (89%)	4 (44%)
PFS overall population	current study	2 (73.5%)	3 (51.1%)	1 (99.8%)	4 (24.7%)
	Riaz et al. (2021)	3 (85.06%)	5 (66.49%)	1 (98.07%)	7 (44.27%)
	Quhal^11^ ^a^	2 (82.16%)	4 (51.63%)	1 (99.06%)	6 (23.72%)
	Nocera^15^ ^a^	4 (39%)	3 (52%)	1 (79%)	2 (64%)
	Bosma et al. (2022)	2 (84%)	4 (56%)	1 (99%)	6 (24%)
PFS IMDC favorable risk population	current study	2 (50.8%)	3 (46.8%)	1 (98.9%)	n.e
	Riaz et al. (2021)	2 (66.45%)	5 (45.64%)	1 (96.37%)	7 (0.12%)
	Quhal^11^ ^a^	2 (59.67%)	4 (58.75%)	1 (92.11%)	6 (10.94%)
	Bosma et al. (2022)	2 (68%)	4 (47%)	1 (96%)	6 (0%)
PFS IMDC intermediate or poor risk population	current study	2 (75.5%)	3 (45,9%)	1 (95.6%)	4 (32.9%)
	Riaz et al. (2021)	2 (78.47%)	5 (41.76%)	1 (95.44%)	6 (31.72%)
	Quhal^11^ ^a^	2 (80.79%)	4 (40.71%)	1 (97.55%)	5 (32.83%)
	Bosma - intermediate Bosma et al. (2022)	2 (74%)	3 (42%)	1 (98%)	n.e
	Bosma - poor Bosma et al. (2022)	2 (75%)	4 (41%)	1 (89%)	n.e

^a^ Quhal and Nocera reported *p*-value instead of SCORE (%); n.e, not estimated; AXY, axitinib; CAB, cabozantinib; IPI, ipilimumab; LEN, lenvatinib; NIV, nivolumab; PEM, pembrolizumab

We conclude that inclusion of the most current cut-off data from CheckMate 9 ER and other comparators does not affect the role of the cabozantinib + nivolumab combination in the list of therapies used for treatment-naïve metastatic clear-cell RCC in all IMDC risk groups. Cabozantinib + nivolumab is an effective therapeutic option for the first-line treatment of advanced renal cancer that is recommended both in the latest European and American guidelines for all IMDC risk groups (Grünwald et al., 2021; Rini et al., 2021; Motzer et al., 2022b).

## Data Availability

The original contributions presented in the study are included in the article/Supplementary Material, further inquiries can be directed to the corresponding author.
