# Supplementary material for: Immune checkpoint inhibitors combined with tyrosine kinase inhibitors or immunotherapy for treatment-naïve metastatic clear-cell renal cell carcinoma—A network meta-analysis. Focus on cabozantinib combined with nivolumab
Source: Front Pharmacol. 2023 Mar 3;13:1063178. doi: 10.3389/fphar.2022.1063178 (PMC10020696; doi:10.3389/fphar.2022.1063178)
Supplement: Supplementary file 1 [file Table1.docx]

# Supplementary Material

## ANNEX 1. The search strategy.

Table I. Search strategy in PubMed, 28/07/2022.

| ID | Query | Records |
| --- | --- | --- |
| #1 | Carcinoma, Nephroid[Text Word] | 5 |
| #2 | Adenocarcinoma Of Kidney[Text Word] | 18 |
| #3 | Adenocarcinoma Of Kidneys[Text Word] | 1 |
| #4 | Kidney, Adenocarcinoma Of[Text Word] | 4 |
| #5 | Renal Cell Carcinoma[Text Word] | 41 877 |
| #6 | Renal Cell Cancer[Text Word] | 3 680 |
| #7 | Cancer, Renal Cell[Text Word] | 284 |
| #8 | Renal Cell Cancers[Text Word] | 231 |
| #9 | Adenocarcinoma, Renal[Text Word] | 43 |
| #10 | Renal Adenocarcinoma[Text Word] | 1 042 |
| #11 | Renal Adenocarcinomas[Text Word] | 124 |
| #12 | Renal Carcinoma[Text Word] | 5 422 |
| #13 | Carcinoma, Renal[Text Word] | 38 397 |
| #14 | Renal Carcinomas[Text Word] | 916 |
| #15 | Adenocarcinoma, Renal Cell[Text Word] | 16 |
| #16 | Adenocarcinomas, Renal Cell[Text Word] | 2 |
| #17 | Renal Cell Adenocarcinoma[Text Word] | 117 |
| #18 | Renal Cell Adenocarcinomas[Text Word] | 12 |
| #19 | #1 OR #2 OR #3 OR #4 OR #5 OR #6 OR #7 OR #8 OR #9 OR #10 OR #11 OR #12 OR #13 OR #14 OR #15 OR #16 OR #17 OR #18 | 56 087 |
| #20 | “cabozantinib” [Supplementary Concept] | 573 |
| #21 | cabozantinib[Text Word] | 1 363 |
| #22 | Cometriq[Text Word] | 9 |
| #23 | XL-184[Text Word] | 26 |
| #24 | BMS907351[Text Word] | 3 |
| #25 | BMS-907351[Text Word] | 5 |
| #26 | #20 OR #21 OR #22 OR #23 OR #24 OR #25 | 1 376 |
| #27 | “Nivolumab”[Mesh] | 4 461 |
| #28 | Nivolumab[Text Word] | 8 326 |
| #29 | Opdivo[Text Word] | 87 |
| #30 | ONO 4538[Text Word] | 21 |
| #31 | ONO4538[Text Word] | 4 |
| #32 | MDX 1106[Text Word] | 5 |
| #33 | MDX1106[Text Word] | 5 |
| #34 | BMS 936558[Text Word] | 24 |
| #35 | BMS936558[Text Word] | 19 |
| #36 | #27 OR #28 OR #29 OR #30 OR #31 OR #32 OR #33 OR #34 OR #35 | 8 339 |
| #37 | “pembrolizumab” [Supplementary Concept] | 3 471 |
| #38 | pembrolizumab[Text Word] | 7 473 |
| #39 | SCH-900475[Text Word] | 2 |
| #40 | Keytruda[Text Word] | 110 |
| #41 | MK-3475[Text Word] | 54 |
| #42 | lambrolizumab[Text Word] | 23 |
| #43 | #37 OR #38 OR #39 OR #40 OR #41 OR #42 | 7 499 |
| #44 | “Axitinib”[Mesh] | 664 |
| #45 | Axitinib[Text Word] | 1 341 |
| #46 | AG013736[Text Word] | 45 |
| #47 | AG-013736[Text Word] | 41 |
| #48 | Inlyta[Text Word] | 16 |
| #49 | #44 OR #45 OR #46 OR #47 OR #48 | 1 350 |
| #50 | “lenvatinib” [Supplementary Concept] | 674 |
| #51 | lenvatinib[Text Word] | 1 591 |
| #52 | Lenvima[Text Word] | 20 |
| #53 | E-7080[Text Word] | 3 |
| #54 | ER-203492-00[Text Word] | 1 |
| #55 | E7080[Text Word] | 57 |
| #56 | #50 OR #51 OR #52 OR #53 OR #54 OR #55 | 1 596 |
| #57 | “Ipilimumab”[Mesh] | 2 640 |
| #58 | Ipilimumab[Text Word] | 4 961 |
| #59 | Anti CTLA 4 MAb Ipilimumab[Text Word] | 6 |
| #60 | Ipilimumab, Anti-CTLA-4 MAb[Text Word] | 2 |
| #61 | Yervoy[Text Word] | 65 |
| #62 | MDX010[Text Word] | 33 |
| #63 | MDX-010[Text Word] | 24 |
| #64 | MDX CTLA 4[Text Word] | 3 |
| #65 | #57 OR #58 OR #59 OR #60 OR #61 OR #62 OR #63 OR #64 | 4 968 |
| #66 | #26 AND #36 | 303 |
| #67 | #43 AND #49 | 162 |
| #68 | #43 AND #56 | 233 |
| #69 | #36 AND #65 | 2 700 |
| #70 | #66 OR #67 OR #68 OR #69 | 3 098 |
| #71 | #19 AND #70 | 648 |
| #72 | randomized controlled trial [pt] | 574 483 |
| #73 | controlled clinical trial [pt] | 664 534 |
| #74 | randomized [tiab] | 620 478 |
| #75 | placebo [tiab] | 236 776 |
| #76 | clinical trials as topic [mesh: noexp] | 375 519 |
| #77 | randomly [tiab] | 388 511 |
| #78 | trial [ti] | 252 546 |
| #79 | #72 OR #73 OR #74 OR #75 OR #76 OR #77 OR #78 | 1 555 436 |
| #80 | animals [mh] NOT humans [mh] | 5 028 691 |
| #81 | #79 NOT #80 | 1 437 851 |
| #82 | #71 AND #81 | 175 |

Table II. Search strategy in EMBASE, 28/07/2022.

| ID | Query | Records |
| --- | --- | --- |
| #1 | ‘kidney cancer’/exp OR ‘kidney cancer’ | 135 312 |
| #2 | ‘cabozantinib’/exp OR ‘cabozantinib’ | 5 953 |
| #3 | ‘nivolumab’/exp OR ‘nivolumab’ | 31 034 |
| #4 | ‘pembrolizumab’/exp OR ‘pembrolizumab’ | 29 309 |
| #5 | ‘axitinib’/exp OR ‘axitinib’ | 6 477 |
| #6 | ‘lenvatinib’/exp OR ‘lenvatinib’ | 4 862 |
| #7 | ‘ipilimumab’/exp OR ‘ipilimumab’ | 21 104 |
| #8 | #2 AND #3 | 1 750 |
| #9 | #4 AND #5 | 1 084 |
| #10 | #4 AND #6 | 1 391 |
| #11 | #3 AND #7 | 13 426 |
| #12 | #8 OR #9 OR #10 OR #11 | 15 087 |
| #13 | #1 AND #12 | 3 414 |
| #14 | #13 AND (‘controlled clinical trial’/de OR ‘controlled study’/de OR ‘phase 2 clinical trial’/de OR ‘phase 3 clinical trial’/de OR ‘randomized controlled trial’/de) | 920 |

## ANNEX 2. The included studies.

Table III. Included studies.

| Study | Methodology | Population | Outcomes |
| --- | --- | --- | --- |
| **Study ID:**  CheckMate 9ER  NCT03141177  **Funding source:**  Bristol Myers Squibb, Ono Pharmaceutical, Exelixis, Ipsen Pharma and Takeda Pharmaceuticals | **Methods:**  Phase 3, prospective, randomized, controlled, open-label, multicenter, parallel study  **Intervention:**  Nivolumab and Cabozantinib  **Comparator:**  Sunitinib  **Time horizon:**  Median: 32.9 months  **Hypothesis:**  Superiority | **Inclusion criteria:**   - Histological confirmation of RCC with a clear-cell component, including participants who may also have sarcomatoid features - Advanced (not amenable to curative surgery or radiation therapy) or metastatic (AJCC Stage IV) RCC - No prior systemic therapy for RCC with the following exception: one prior adjuvant or neoadjuvant therapy for completely resectable RCC if such therapy did not include an agent that targets VEGF or VEGF receptors and if recurrence occurred at least 6 months after the last dose of adjuvant or neoadjuvant therapy   **Exclusion criteria:**   - Any active CNS metastases - Any active, known or suspected autoimmune disease - Any condition requiring systemic treatment with either corticosteroids (> 10 mg daily prednisone equivalent) or other immunosuppressive medications within 14 days of randomization - Participants who have received a live/attenuated vaccine within 30 days of first treatment   **Number of patients included:**  N = 651 (ITT)  Intervention: N = 323  Comparator: N = 328 | **Primary:**  PFS  **Other:**  OS  ORR  Safety  QoL |
| **Study ID:**  CheckMate 214  **Funding source:**  Bristol-Myers Squibb and Ono Pharmaceutical | **Methods:**  Phase 3, prospective, randomized, controlled, open-label, multicenter, parallel study  **Intervention:**  Nivolumab and Ipilimumab  **Comparator:**  Sunitinib  **Time horizon:**  Median: 67.7 months  **Hypothesis**  Superiority | **Inclusion criteria:**   - Histological confirmation of RCC with a clear-cell component - Advanced (not amenable to curative surgery or radiation therapy) or metastatic (AJCC Stage IV) RCC - No prior systemic therapy for RCC with the following exception: one prior adjuvant or neoadjuvant therapy for completely resectable RCC if such therapy did not include an agent that targets VEGF or VEGF receptors and if recurrence occurred at least 6 months after the last dose of adjuvant or neoadjuvant therapy - KPS of at least 70% - Measurable disease as per RECIST 1.1   **Exclusion criteria:**   - Any history of or current CNS metastases. Baseline imaging of the brain is required within 28 days prior to randomization - Prior systemic treatment with VEGF or VEGF receptor targeted therapy - Prior treatment with an anti-programmed death (PD)-1, anti- programmed cell death ligand 1 (PD-L1), anti-PD-L2, anti-CD137, or anti-cytotoxic T-lymphocyte antigen 4 (CTLA-4) antibody, or any other antibody or drug specifically targeting T-cell co-stimulation or checkpoint pathways - Any condition requiring systemic treatment with corticosteroids (>10 mg daily prednisone equivalents) or other immunosuppressive medications within 14 days prior to first dose of study drug. Inhaled steroids and adrenal replacement steroid doses >10 mg daily   **Number of patients included:**  N = 1 096 (ITT)  Intervention: N = 550  Comparator: N = 546 | **Primary:**  PFS  OS  ORR (intermediate/poor prognosis population)  **Other:**  PFS  OS  ORR (ITT)  Safety  QoL |
| **Study ID:**  CLEAR  **Funding source:**  Eisai, Merck, Sharp and Dohme | **Methods:**  Phase 3, prospective, randomized, controlled, open-label, multicenter, parallel study  **Intervention:**  Lenvatinib and Everolimus  Lenvatinib and Pembrolizumab  **Comparator:**  Sunitinib  **Time horizon:**  Median: 26.6 months  **Hypothesis**  Superiority | **Inclusion criteria:**   - Histological or cytological confirmation of RCC with a clear-cell component - At least 1 measurable target lesion according to RECIST 1.1 - KPS of ≥ 70 - Adequately controlled blood pressure (BP) with or without antihypertensive medications, defined as BP ≤ 150/90 mmHg at screening and no change in antihypertensive medications within 1 week prior to Cycle 1/Day 1 (C1/D1) - Adequate organ function per blood work   **Exclusion criteria:**   - Any systemic anticancer therapy for RCC, including anti-VEGF therapy or any systemic investigational anticancer agent - CNS metastases, unless patients have completed local therapy and have discontinued the use of corticosteroids for this indication for at least 4 weeks before starting treatment in this study - Active malignancy (except for RCC, definitively treated basal or squamous cell carcinoma of the skin, and carcinoma in-situ of the cervix or bladder) within the past 24 months - Prior radiation therapy within 21 days prior to start of study treatment with the exception of palliative radiotherapy to bone lesions, which is allowed if completed 2 weeks prior to study treatment start   **Number of patients included:**  N = 712 (ITT)  Intervention: N = 355  Comparator: N = 357 | **Primary:**  PFS  **Other:**  OS  ORR  Duration of response to treatment  Safety |
| **Study ID:**  KEYNOTE-426  **Funding source:**  Merck, Sharp and Dohme | **Methods:**  Phase 3, prospective, randomized, controlled, open-label, multicenter, parallel study  **Intervention:**  Pembrolizumab and Axitinib  **Comparator:**  Sunitinib  **Time horizon:**  Median: 42.8 months  **Hypothesis**  Superiority | **Inclusion criteria:**   - Histologically confirmed diagnosis of RCC with clear cell component with or without sarcomatoid features - Locally advanced/metastatic disease (i.e., newly diagnosed Stage IV RCC per AJCC) or recurrent disease - Measurable disease per RECIST 1.1 as assessed by the investigator/site radiologist. - No prior systemic therapy for advanced RCC - KPS ≥ 70% as assessed within 10 days prior to randomization - If receiving bone resorptive therapy (including but not limited to bisphosphonate or RANK-L inhibitor) must have therapy initiated at least 2 weeks prior to randomization - Adequate organ function   **Exclusion criteria:**   - Major surgery within 4 weeks, received radiation therapy within 2 weeks prior to randomization, or has not recovered (i.e., ≤ Grade 1 or at baseline) from AEs due to prior treatment - Prior treatment with any anti-PD-1, PD-L1, or PD-L2 agent or an antibody targeting any other immune-regulatory receptors or mechanisms - Prior systemic anti-cancer therapy for RCC with VEGF/VEGF receptors or mechanistic target of rapamycin (mTOR) targeting agents - Known active CNS metastases and/or carcinomatous meningitis   **Number of patients included:**  N = 861 (ITT)  Intervention: N = 432  Comparator: N = 429 | **Primary:**  PFS  OS  **Other:**  ORR  Duration of response to treatment  Safety |
| AJCC — American Joint Committee on Cancer; CNS — central nervous system; ITT — intention to treat; KPS — Karnofsky Performance Status; ORR — objective response rate; OS —overall survival; PFS — progression free survival; RCC — renal cell carcinoma; RECIST — Response Evaluation Criteria in Solid Tumors; QoL – quality of life; VEGF — vascular endothelial growth factor. | | | |

## ANNEX 3. The excluded studies.

Table IV. Studies excluded from the analysis.

| **Study ID** | **Reference** | **Reasons for exclusion** |
| --- | --- | --- |
| Tamada 2021 | Tamada S, Kondoh C, Matsubara N, Mizuno R, Kimura G, Anai S, Tomita Y, Oyama M, Masumori N, Kojima T, Matsumoto H, Chen M, Li M, Matsuda K, Tanaka Y, Rini BI, Uemura H. Pembrolizumab plus axitinib versus sunitinib in metastatic renal cell carcinoma: outcomes of Japanese patients enrolled in the randomized, phase III, open-label KEYNOTE-426 study. Int J Clin Oncol. 2021 Nov 20. doi: 10.1007/s10147-021-02014-7. | The publication regards patients recruited from Japan-based centers only.  Lack of new results for the overall population. |
| Regan 2021 | Regan MM, Jegede OA, Mantia CM, Powles T, Werner L, Motzer RJ, Tannir NM, Lee CH, Tomita Y, Voss MH, Plimack ER, Choueiri TK, Rini BI, Hammers HJ, Escudier B, Albiges L, Huo S, Del Tejo V, Stwalley B, Atkins MB, McDermott DF. Treatment-free Survival after Immune Checkpoint Inhibitor Therapy versus Targeted Therapy for Advanced Renal Cell Carcinoma: 42-Month Results of the CheckMate 214 Trial. | The publication regards analysis of the survival duration without treatment.  Results are not reported as hazard ratios. |
| Albiges 2021 | Albiges L, Tannir NM, Burotto M, McDermott D, Plimack ER, Barthélémy P, Porta C, Powles T, Donskov F, George S, Kollmannsberger CK, Gurney H, Grimm MO, Tomita Y, Castellano D, Rini BI, Choueiri TK, Leung D, Saggi SS, Lee CW, McHenry MB, Motzer RJ. First-line Nivolumab plus Ipilimumab Versus Sunitinib in Patients Without Nephrectomy and With an Evaluable Primary Renal Tumor in the CheckMate 214 Trial. Eur Urol. 2021 Nov 5:S0302-2838(21)02069-8. | Analysis of the selected subpopulation of patients from CheckMate 214 study.  Lack of new results for the overall population. |
| Rini 2021 | Rini BI, Atkins MB, Plimack ER, Soulières D, McDermott RS, Bedke J, Tartas S, Alekseev B, Melichar B, Shparyk Y, Kondoh C, Langiewicz P, Wood LA, Hammers H, Silber CG, Haber B, Jensen E, Chen M, Powles T. Characterization and Management of Treatment-emergent Hepatic Toxicity in Patients with Advanced Renal Cell Carcinoma Receiving First-line Pembrolizumab plus Axitinib. Results from the KEYNOTE-426 Trial. Eur Urol Oncol. 2021 Jul 6:S2588-9311(21)00113-9. | Only the data on liver toxicity occurring during treatment with pembrolizumab + axitinib was analyzed in the publication.  Lack of new results for the overall population. |
| Schmidinger 2021 | Schmidinger M, Shariat SF, Fajkovic H. Dual immune checkpoint inhibition in metastatic renal cell carcinoma: Editorial re.: Nivolumab plus ipilimumab versus sunitinib for first-line treatment of advanced RCC: extended 4-year follow-up of the phase III CheckMate 214 trial. ESMO Open. 2021 Feb;6(1):100035. | Commentary on Albiges 2020, no additional results. |
| Tannir 2021 | Tannir NM, Signoretti S, Choueiri TK, McDermott DF, Motzer RJ, Flaifel A, Pignon JC, Ficial M, Frontera OA, George S, Powles T, Donskov F, Harrison MR, Barthélémy P, Tykodi SS, Kocsis J, Ravaud A, Rodriguez-Cid JR, Pal SK, Murad AM, Ishii Y, Saggi SS, McHenry MB, Rini BI. Efficacy and Safety of Nivolumab Plus Ipilimumab versus Sunitinib in First-line Treatment of Patients with Advanced Sarcomatoid Renal Cell Carcinoma. Clin Cancer Res. 2021 Jan 1;27(1):78-86. doi: 10.1158/1078-0432.CCR-20-2063. | The publication regards the results for a selected group of patients (i.e., patients with renal cell sarcoma). Lack of new results for the overall population. |
| Choueiri 2021 | Choueiri T.K., Eto M., Kopyltsov E., Rha S.Y., Porta C.G., Motzer R., Grünwald V., Hutson T.E., Méndez-Vidal M.J., Hong S.-H., Winquist E., Goh J.C.H., Maroto P., Buchler T., Takagi T., Rodriguez-Lopez K., Xing D., Smith A.D., Powles T. Phase III CLEAR trial in advanced renal cell carcinoma (aRCC): Outcomes in subgroups and toxicity update. Annals of Oncology (2021) 32 Supplement 5 (S683-S685). | Lack of new results for the overall population or subpopulations by risk categories. |
| Gafanov 2021 | Gafanov R., Powles T.B., Bedke J., Stus V., Waddell T.S., Nosov D., Pouliot F., Soulieres D., Melichar B., Azevedo S., McDermott R.S., Vynnychenko I.O., Borchiellini D., Markus M., Bondarenko I., Lin J., Burgents J., Molife L.R., Plimack E.R., Rini B. Subsequent therapy following pembrolizumab + axitinib or sunitinib treatment for advanced renal cell carcinoma (RCC) in the phase III KEYNOTE-426 study. Annals of Oncology (2021) 32 Supplement 5 (S694). | Lack of new data regarding overall survival and progression-free survival as compared to the main publications. |
| Spisarová 2021 | Spisarová M, Melichar B, Vitásková D, Študentová H. Pembrolizumab plus axitinib for the treatment of advanced renal cell carcinoma. Expert Rev Anticancer Ther. 2021 Jul;21(7):693-703. | Lack of new data as compared to the main publications for the KEYNOTE-426 study. |
| Motzer 2021a | Motzer R, Alekseev B, Rha SY, et al. Lenvatinib Plus Pembrolizumab Improves Outcomes in Previously Untreated Advanced Clear Cell Renal Cell Carcinoma. March/April 2021 Vol. 28, No. 2 | Lack of new data as compared to the main publication. |
| Motzer 2021b | Motzer R.J., Choueiri T.K., Powles T., Burotto M., Bourlon M.T., Hsieh J.J., Maruzzo M., Shah A.Y., Suarez C., Barrios C.H., Richardet M.E., Porta C., Goh J.C., Tomita Y., Bedke J., Zhang J., Simsek B., Scheffold C., Gupta S., Apolo A.B. Nivolumab + cabozantinib (NIVO+CABO) versus sunitinib (SUN) for advanced renal cell carcinoma (aRCC): Outcomes by sarcomatoid histology and updated trial results with extended follow-up of CheckMate 9ER. Journal of Clinical Oncology (2021) 39:6 SUPPL. | Lack of new data for the overall population.  Only the results for patients with sarcoid renal cell carcinoma are discussed in the abstract. |
| Tannir 2021a | Tannir N.M., Motzer R.J., Albiges L., Plimack E.R., George S., Powles T., Donskov F., Rini B.I., Grünwald V., Hammers H.J., Choueiri T.K., Gurney H., Tykodi S.S., Porta C., Burotto M., Tomita Y., Lee C.-W., Tang C., McDermott D.F., McKay R.R. Patterns of progression in patients treated with nivolumab plus ipilimumab (NIVO+IPI) versus sunitinib (SUN) for first-line treatment of advanced renal cell carcinoma (aRCC) in CheckMate 214. Journal of Clinical Oncology (2021) 39:6 SUPPL. | Lack of new data regarding overall survival and progression-free survival as compared to the main publications. |
| Motzer 2021c | Motzer R.J., Porta C., Eto M., Powles T., Grünwald V., Hutson T.E., Alekseev B., Rha S.Y., Kopyltsov E., Vidal M.J.M., Hong S.-H., Kapoor A., Gordoa T.A., Goh J.C., Merchan J.R., Smith A.D., Mody K., Perini R.F., Xing D., Choueiri T.K. Phase 3 trial of lenvatinib (LEN) plus pembrolizumab (PEMBRO) or everolimus (EVE) versus sunitinib (SUN) monotherapy as a first-line treatment for patients (pts) with advanced renal cell carcinoma (RCC) (CLEAR study). Journal of Clinical Oncology (2021) 39:6 SUPPL. | Lack of new data regarding overall population, as well as subpopulations by risk category, as compared to the main publications |
| Motzer 2021d | Motzer R.J., Choueiri T.K., May J., Kwon Y., Rusibamayila N., Botteman M., Hamilton M., Ejzykowicz F., Cella D. Long-term trend of quality adjusted time without symptoms or toxicities (Q-TWiST) of nivolumab+ipilimumab (N+I) versus sunitinib (SUN) for the first-line treatment of advanced renal cell carcinoma (aRCC). Journal of Clinical Oncology (2021) 39:15 SUPPL. Date of Publication: 2021 | Lack of new data regarding overall survival and progression-free survival as compared to the main publications. |
| Shah 2021 | Shah A.Y., Motzer R.J., Apolo A.B., Powles T., Escudier B., Zhang J., Scheffold C., Karumanchi S., Nguyen L.T., Choueiri T.K. Cabozantinib (C) exposure-response (ER) analysis for the phase 3 Check Mate 9ER (CM 9ER) trial of nivolumab plus cabozantinib (N+C) versus sunitinib (S) in first-line advanced renal cell carcinoma (1L aRCC). Journal of Clinical Oncology (2021) 39:15 SUPPL. Date of Publication: 2021 | Lack of new data regarding overall population as compared to the main publications. |

## ANNEX 4. Networks used in the analysis.

Figure I. Network used for subpopulations with a favorable prognosis (overall survival and progression-free survival).


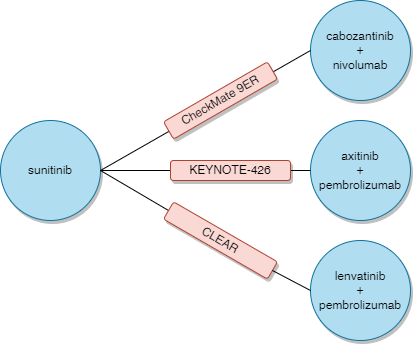


Figure II. Network used for subpopulations with an intermediate/poor prognosis (overall survival and progression-free survival).


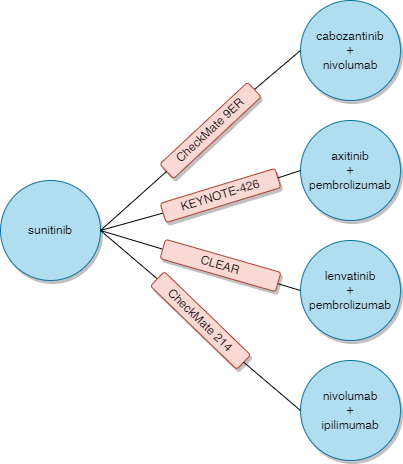


## ANNEX 5. Results extracted from individual studies included in the review.

Table V. Overall survival results extracted from individual studies included in the analysis.

| **Study Id** | **Comparison** | **Data cut-off date/median observation period** | **Hazard Ratio (95% CI)** | **Source** |
| --- | --- | --- | --- | --- |
| **Overall population** | | | | |
| CheckMate 9ER | Cabozantinib + nivolumab vs. sunitinib | June 24, 2021/32.9 months | 0.70 (0.55– 0.90) | Motzer 2022^[[1]](#endnote-2)^ |
| KEYNOTE-426 | Axitinib + pembrolizumab vs. sunitinib | 42.8 months | 0.73 (0.60–0.88) | Conference abstract: Rini 2021^[[2]](#endnote-3)^ |
| CLEAR | Lenvatinib + pembrolizumab vs. sunitinib | August 28, 2020/ 26.6 months | 0.66 (0.49– 0.88) | Main publication: Motzer 2021^10^ |
| CheckMate 214 | Nivolumab + ipilimumab vs. sunitinib | 67.7 months | 0.72 (0.62– 0.85) | Motzer 2022b^19^ |
| **Subpopulation of patients with a favorable prognosis** | | | | |
| CheckMate 9ER | Cabozantinib + nivolumab vs. sunitinib | June 24, 2021/32.9 months | 1.03 (0.55–1.92) | Motzer 2022^31^ |
| KEYNOTE-426 | Axitinib + pembrolizumab vs. sunitinib | 42.8 months | 1.17 (0.76– 1.80) | Conference abstract: Rini 2021^32^ |
| CLEAR | Lenvatinib + pembrolizumab vs. sunitinib | August 28, 2020/ 26.6 months | 1.15 (0.55– 2.40) | Appendix to the main publication: Motzer 2021^10^ |
| **Subpopulation of patients with an intermediate/poor prognosis** | | | | |
| CheckMate 9ER | Cabozantinib + nivolumab vs. sunitinib | June 24, 2021/32.9 months | 0.63 (0.42– 0.93) | Meta-analysis of the results for the subgroup of intermediate/poor prognosis pts, Motzer 2022^31^ |
| KEYNOTE-426 | Axitinib + pembrolizumab vs. sunitinib | 42.8 months | 0.64 (0.52– 0.80) | Conference abstract: Rini 2021^32^ |
| CLEAR | Lenvatinib + pembrolizumab vs. sunitinib | August 28, 2020/ 26.6 months | 0.58 (0.42– 0.80) | Conference abstract: Grünwald 2021^[[3]](#endnote-4)^ |
| CheckMate 214 | Nivolumab + ipilimumab vs. sunitinib | 67.7 months | 0.68 (0.58– 0.81) | Motzer 2022b^19^ |

Figure III. Overall survival for the subpopulation of patients with an intermediate/poor prognosis in CheckMate 9ER. Results from a meta-analysis of the data.


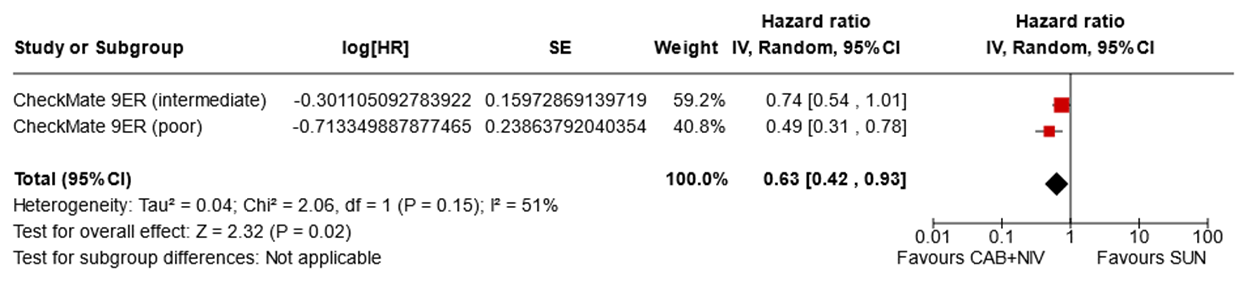


Table VI. Progression-free survival results extracted from individual studies included in the analysis.

| **Study** | **Comparison** | **Data cut-off date/median observation period** | **Hazard Ratio (95% CI)** | **Source** |
| --- | --- | --- | --- | --- |
| **Overall population** | | | | |
| CheckMate 9ER | Cabozantinib + nivolumab vs. sunitinib | June 24, 2021/32.9 months | 0.56 (0.46– 0.68) | Motzer 2022^29^ |
| KEYNOTE-426 | Axitinib + pembrolizumab vs. sunitinib | 42.8 months | 0.68 (0.58– 0.80) | Conference abstract: Rini 2021^32^ |
| CLEAR | Lenvatinib + pembrolizumab vs. sunitinib | August 28, 2020/ 26.6 months | 0.39 (0.32– 0.49) | Main publication: Motzer 2021^10^ |
| CheckMate 214 | Nivolumab + ipilimumab vs. sunitinib | 67.7 months | 0.86 (0.73– 1.01) | Motzer 2022b^19^ |
| **Subpopulation of patients with a favorable prognosis** | | | | |
| CheckMate 9ER | Cabozantinib + nivolumab vs. sunitinib | June 24, 2021/32.9 months | 0.73 (0.48– 1.11) | Motzer 2022^29^ |
| KEYNOTE-426 | Axitinib + pembrolizumab vs. sunitinib | 42.8 months | 0.76 (0.56– 1.03) | Conference abstract: Rini 2021^32^ |
| CLEAR | Lenvatinib + pembrolizumab vs. sunitinib | August 28, 2020/ 26.6 months | 0.41 (0.28– 0.62) | Main publication: Motzer 2021^10^ |
| **Subpopulation of patients with intermediate/poor prognosis** | | | | |
| CheckMate 9ER | Cabozantinib + nivolumab vs. sunitinib | June 24, 2021/32.9 months | 0.47 (0.28– 0.78) | Meta-analysis of the results for the subgroup of intermediate/poor prognosis pts, conference abstracts: Motzer 2022^29^ |
| KEYNOTE-426 | Axitinib + pembrolizumab vs. sunitinib | 42.8 months | 0.67 (0.55– 0.81) | Conference abstract: Rini 2021^32^ |
| CLEAR | Lenvatinib + pembrolizumab vs. sunitinib | August 28, 2020/ 26.6 months | 0.36 (0.28– 0.47) | Conference abstract: Grünwald 2021^33^ |
| CheckMate 214 | Nivolumab + ipilimumab vs. sunitinib | 67.7 months | 0.73 (0.61– 0.87) | Motzer 2022b^19^ |

Figure IV. Progression-free survival for the subpopulation of patients with an intermediate/poor prognosis in CheckMate 9ER. Results from a meta-analysis of the data.


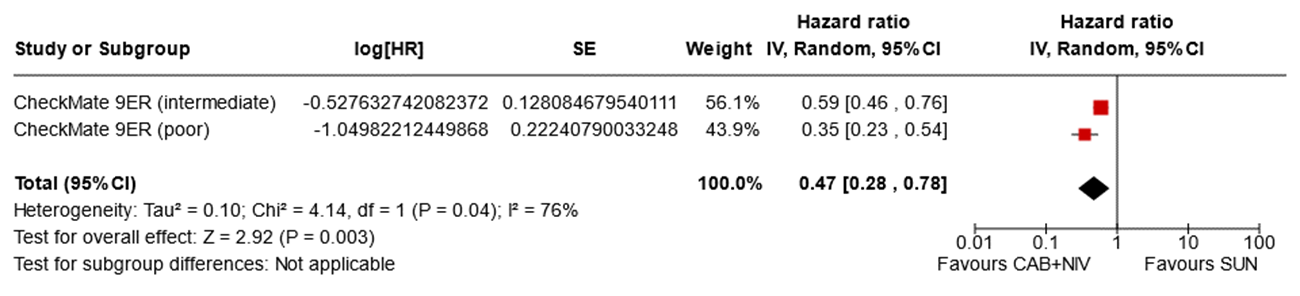


1. [↑](#endnote-ref-2)
2. [↑](#endnote-ref-3)
3. [↑](#endnote-ref-4)
